# Ferrostatin-1 Prevents Salivary Gland Dysfunction in an Ovariectomized Rat Model by Suppressing Mitophagy-Driven Ferroptosis

**DOI:** 10.3390/antiox14091058

**Published:** 2025-08-28

**Authors:** Gi Cheol Park, Soo-Young Bang, Ji Min Kim, Sung-Chan Shin, Yong-il Cheon, Hanaro Park, Sunghwan Suh, Jung Hwan Cho, Eui-Suk Sung, Minhyung Lee, Jin-Choon Lee, Byung-Joo Lee

**Affiliations:** 1 Department of Otolaryngology—Head and Neck Surgery, Samsung Changwon Hospital, Sungkyunkwan University School of Medicine, Changwon 51353, Republic of Korea; uuhent@skku.edu (G.C.P.); naronaro@skku.edu (H.P.); 2 Department of Otorhinolaryngology—Head and Neck Surgery, College of Medicine, Pusan National University and Biomedical Research Institute, Pusan National University Hospital, Busan 49241, Republic of Korea; sooyoungbang@pusan.ac.kr (S.-Y.B.); jimin-kim@pusan.ac.kr (J.M.K.); shinsc0810@pusan.ac.kr (S.-C.S.); skydragonone@pusan.ac.kr (Y.-i.C.); 3 Divisions of Endocrinology and Metabolism, Department of Internal Medicine, Samsung Changwon Hospital, Sungkyunkwan University School of Medicine, Changwon 51353, Republic of Korea; suhs@skku.edu (S.S.);; 4 Department of Otorhinolaryngology—Head and Neck Surgery, College of Medicine, Pusan National University and Biomedical Research Institute, Pusan National University Yangsan Hospital, Yangsan 50613, Republic of Korea; sunges@pusan.ac.kr (E.-S.S.); lee.minhyung.ent@gmail.com (M.L.); ljc020971@pusan.ac.kr (J.-C.L.); 5 Department of Otorhinolaryngology—Head and Neck Surgery, Good Gang—An Hospital, Busan 48265, Republic of Korea

**Keywords:** menopause, ferroptosis, salivary gland dysfunction, mitochondria, mitophagy

## Abstract

Salivary gland dysfunction is a common but underexplored complication of menopause that contributes to oral dryness, dysphagia, and increased risk of infection. Although ferroptosis, a form of regulated necrotic cell death driven by iron-dependent lipid peroxidation, has recently been implicated in postmenopausal tissue degeneration, its regulatory mechanisms in salivary glands remain unclear. In this study, we investigated the roles of mitochondrial dysfunction and mitophagy in driving ferroptosis-induced salivary gland injury in an ovariectomized (OVX) rat model of estrogen deficiency. OVX rats exhibited elevated markers of oxidative stress, lipid accumulation, and iron overload, and suppression of GPX4 activity in the salivary glands, consistent with ferroptotic activation. These changes were accompanied by impaired mitochondrial dynamics (MFN1 and OPA1), decreased expression of mitochondrial antioxidant regulators (PGC-1α, SOD, and catalase), and upregulation of mitophagy-related genes (*PINK1*, *ULK1*, *Rab9*, and *LC3B*), as well as LAMP, a lysosomal marker involved in autophagosome–lysosome fusion, while ferritinophagy (NCOA4) remained unchanged. Early administration of ferrostatin-1 effectively suppressed these pathological changes, preserving both glandular structure and function, as evidenced by the restored AQP5 and AMY2A expression. Collectively, our findings reveal that ferroptosis in estrogen-deficient salivary glands is regulated by mitochondrial instability and aberrant mitophagy, and ferrostatin-1 mitigates this cascade through multi-level mitochondrial protection. These results highlight ferrostatin-1 as a promising preventive agent against menopause-associated salivary gland dysfunction, with broader implications for organ-specific ferroptosis modulation.

## 1. Introduction

Menopause induces a variety of physiological changes that substantially affect the quality of life of women, and salivary gland dysfunction is recognized as a major complication [1,2,3]. Estrogen deficiency leads to salivary gland atrophy, reduced salivary secretion, fibrosis, and inflammatory responses, contributing to symptoms such as xerostomia, dysphagia, and an increased risk of oral infections in postmenopausal women [4,5,6]. However, the cellular and molecular mechanisms underlying menopause-related salivary gland dysfunction remain largely unclear, and effective preventive or therapeutic strategies are still limited.

Ferroptosis is an iron-dependent form of regulated cell death characterized by lipid peroxidation, depletion of glutathione peroxidase 4 (GPX4), and accumulation of reactive oxygen species (ROS). It has been implicated in a wide range of diseases, including neurodegeneration, cancer, ischemic injury, and hepatic fibrosis [7,8,9]. Recent studies have shown that ferroptosis is regulated not only by iron metabolism but also by selective autophagic pathways such as ferritinophagy (via NCOA4) and mitophagy, which modulate intracellular iron homeostasis and mitochondrial function, respectively [10,11]. Although these upstream regulators are increasingly recognized in other organs, their relative contribution to ferroptosis in the salivary glands remains unclear, particularly in the setting of estrogen deficiency.

In our previous study using an ovariectomized (OVX) rat model, we demonstrated that treatment with ferrostatin-1, a ferroptosis inhibitor, alleviated oxidative stress, fibrosis, and secretory dysfunction in estrogen-deficient salivary glands [12,13]. However, that study primarily focused on terminal ferroptosis markers, such as GPX4 depletion and lipid peroxidation, and did not explore the upstream molecular regulators that initiate or modulate ferroptotic injury. In a separate study using hepatic tissue, we identified NCOA4-mediated ferritinophagy as a key ferroptosis pathway in the liver [14], but it is unknown whether the same mechanism operates in salivary glands, which differ in iron storage capacity, mitochondrial density, and oxidative vulnerability.

Given the central role of mitochondria in redox regulation and cell death, we hypothesized that mitochondrial dysfunction, mitophagy activation, and impaired mitochondrial antioxidant defense may represent important upstream triggers of ferroptosis in estrogen-deficient salivary glands. In particular, pathways involving PINK1, ULK1, Rab9, and LC3B (mitophagy), MFN1 and OPA1 (mitochondrial dynamics), and PGC-1α, SOD, and catalase (antioxidant regulation) may differ from ferritinophagy-driven ferroptosis in other tissues. Understanding these organ-specific pathways is crucial for developing targeted interventions for postmenopausal glandular degeneration [15,16,17].

Therefore, in the present study, we aimed to investigate the mitochondrial-level regulatory mechanisms of ferroptosis in the salivary glands of OVX rats, with a particular focus on mitophagy and mitochondrial quality control. By extending the scope of our previous work, this study provides a more comprehensive understanding of how ferroptosis is modulated in salivary tissue and highlights novel targets for the prevention of menopause-associated salivary gland dysfunction.

## 2. Materials and Methods

### 2.1. Ovariectomized Rat Model

Eight-week-old female Sprague–Dawley rats were purchased from Central Laboratory Animals Inc. (Seoul, Republic of Korea). To induce menopause, the rats were allowed to adapt for 1 week. Rats do not naturally undergo menopause; thus, a menopausal model was established by bilateral ovariectomy. Rats were randomly divided into three groups: SHAM (*n* = 8, sham-operated rats), OVX (*n* = 8, ovariectomized rats), and FER group (*n* = 8, ovariectomized rats injected with ferrostatin-1).

For ovariectomy, the rats were anesthetized using isoflurane inhalation (3% dissolved in oxygen), and the skin was incised through a bilateral transverse flank incision on the abdomen. In the ovariectomized (OVX) group, the ovaries were ligated and resected bilaterally, and the abdominal cavity was closed. In the SHAM group, the ovaries were exposed without ovariectomy. Ovariectomized rats were intraperitoneally injected with 5 μM/kg of ferrostatin-1 (FER) three times a week for 8 weeks. Drug injections were administered after a one-week recovery period following oophorectomy. The ferroptosis inhibitor, ferrostatin-1, was purchased from Sigma–Aldrich (St. Louis, MO, USA).

After 8 weeks of drug injection, the animals were anesthetized using isoflurane gas (2–3%) delivered via a nose cone, and blood was collected from the inferior vena cava for biomarker testing. Subsequently, they were sacrificed.

The study was approved by the Institutional Animal Care and Ethics Committee of Pusan National University Hospital (No. PNUH-2021-179).

### 2.2. Lipid Peroxidation

Lipid peroxidation induces the production of reactive aldehydes, including malondialdehyde (MDA), which serves as an important indicator of oxidative stress and can be detected using an MDA assay kit (Abcam, Cambridge, UK). The lipid peroxidation assay kit reacts the MDA in the sample with thiobarbituric acid (TBA) to form MDA-TBA adducts. For evaluation of salivary gland tissues, the samples and standards were homogenized in TBA solution, incubated at 95 °C for 60 min, and cooled in an ice bath for 10 min. The MDA-TBA adducts were quantified and analyzed using a colorimetric method (OD = 532 nm).

### 2.3. Cytosolic Iron Assay

Approximately 30 mg of tissue was collected for each assay and briefly rinsed in cold phosphate-buffered saline (PBS) to remove residual blood and extracellular contaminants. The tissue was then transferred to a pre-chilled homogenization vessel and suspended in 300 µL of iron assay buffer. Homogenization was performed using a Dounce homogenizer (Corning Inc., New York, NY, USA) with 30–50 strokes to ensure complete mechanical disruption, while maintaining the samples on ice to prevent enzymatic degradation. Cytoplasmic fractions were obtained by centrifugation at 16,000× *g* for 10 min at 4 °C. Iron concentrations were measured according to the manufacturer’s instructions for the iron assay kit, calculated from standard curves, and normalized to total protein content determined using the bicinchoninic acid (BCA) assay (Thermo Fisher Scientific, Waltham, MA, USA).

### 2.4. ROS Measurement

Reactive oxygen species (ROS) levels in salivary gland tissue were quantified using the fluorescent probe 2′,7′-dichlorodihydrofluorescein diacetate (DCFH-DA), as described in the revised Section 2. Approximately 30 mg of tissue was homogenized in phosphate-buffered saline (PBS), and the supernatant was collected after centrifugation. The DCFH-DA probe was then added to the homogenate at a final concentration of 25 µM in a total volume of 250 µL. Fluorescence intensity, which is directly correlated with ROS production, was recorded at 5 min intervals over a 30 min period using a fluorescence plate reader (BMG LABTECH, Ortenberg, Germany) (excitation: 485 nm; emission: 530 nm). Protein concentrations were determined using the bicinchoninic acid (BCA) assay (Thermo Fisher Scientific, Waltham, MA, USA), with bovine serum albumin (BSA; Sigma-Aldrich, St. Louis, MO, USA) as the standard.

### 2.5. GPX4 Activity

GPX4 activity was assayed using a Glutathione Peroxidase Assay Kit (Abcam), which measured the activity of glutathione peroxidase using the decrease in NADPH absorbance. The protein content of the samples was measured relative to bovine serum albumin standards (Sigma-Aldrich) using the BCA assay.

### 2.6. Staining and Immunohistochemistry Analysis

Salivary glands were isolated from each rat, fixed overnight in 4% formalin, and embedded in paraffin. Cross-sections of the salivary glands were placed on glass slides, and sections were prepared for hematoxylin–eosin (H&E) and Masson’s trichrome staining, and immunohistochemistry. For quantitative analysis of expression in salivary gland samples, deparaffinized sections were incubated with primary antibodies for 24 h at 4 °C. After removing the primary antibodies and washing, the sections were incubated with goat anti-rabbit secondary antibody (ENZO Biochem Inc., New York, NY, USA) for 1 h at room temperature and double-stained with DAB (3,3-diaminobenzidine). Incubation with phosphate-buffered saline supplemented with 1% bovine serum albumin instead of primary antibodies was used as a negative control. We selected the appropriate central part of the tissue for representative images captured at 200× magnification using a light microscope (Leica, Wetzlar, Germany).

### 2.7. Lipid Vacuole Quantification

Lipid vacuoles were quantified from hematoxylin and eosin (H&E)-stained salivary gland sections by analyzing the entire stained section for each sample. Digital images covering the whole tissue section were captured at 200× magnification using a Leica light microscope under identical illumination and exposure settings. Image analysis was performed with ImageJ 1.53e software (NIH, Bethesda, MD, USA). Vacuole-like unstained regions within the acinar cytoplasm were identified by threshold adjustment, and only vacuoles with an area ≥ 50 µm^2^ were counted to exclude small non-specific spaces or artifacts. Counts from the entire section were summed to obtain the total number of lipid vacuoles per sample. No field selection or partial sampling was used.

### 2.8. Electron Microscopy

Tissues were pre-fixed with 2.5% glutaraldehyde (4 °C, phosphate buffer, pH 7.4) for more than 24 h, and then post-fixed with 1% osmium tetroxide in the same buffer. The samples were dehydrated with a series of graded ethyl alcohol solutions and embedded in epoxy resin (Epon 812 mixture). Thick sections (1 μm) were stained with 1% toluidine blue for imaging under a light microscope. Thin sections (50–60 nm) were prepared using an ultramicrotome (EM UC7; Leica) and double-stained with uranyl acetate and lead citrate. The morphological characteristics of the mitochondria were observed using electron microscopy (JEM-1200EXII, JEL Ltd., Tokyo, Japan).

### 2.9. Real-Time Quantitative Polymerase Chain Reaction (RT-qPCR)

Total RNA from the OVX, FER treatment, and control groups was extracted using TRIzol reagent. Complementary DNA (cDNA) was synthesized using the reverse transcription kit (Applied Biosystems, Foster City, CA, USA) at 42 °C for 60 min and 95 °C for 10 min according to the manufacturer’s protocol. The cDNA was subjected to RT-qPCR analysis using SYBR Green PCR. Gene-specific PCR products were continuously measured using an ABI PRISM 7900 HT Sequence Detection System (Applied Biosystems). GAPDH was used as a standard control to analyze the relative mRNA expression levels according to the 2^(−ΔΔCt)^ method. The primer sequences are listed in Table 1.

### 2.10. Western Blotting

Salivary gland tissue immersed in RIPA buffer was ground using a precooled tissue homogenizer, lysed in an ice bath, and centrifuged at 12,000× *g* for 15 min. All protein samples were subjected to sodium dodecyl sulfate–polyacrylamide gel electrophoresis and transferred onto nitrocellulose blotting membranes (Amersham Pharmacia Biotech, Amersham, UK), followed by blocking at room temperature for 1 h. The membrane was incubated overnight at 4 °C with primary antibodies: LC3B 1:1000; LAMP2 1:1000; phospho-ULK1 1:1000; RAB9A 1:1000; and β-actin. After washing five times with TBST, the membranes were incubated with horseradish peroxidase-labeled secondary antibodies (1:5000) for 2 h at room temperature (22 °C). Membranes were washed five times with TBST and developed using an ECL kit (Amersham Pharmacia Biotech).

### 2.11. Statistical Analysis

The data are expressed as the mean ± standard deviation in all experiments. One-way analysis of variance (SPSS version 19.0 software; SPSS, Inc., Chicago, IL, USA), followed by Scheffé’s test, was conducted to detect significant differences between groups. A *p*-value of <0.05 was considered statistically significant.

## 3. Results

### 3.1. Body Weight, Food Intake, and Serum Glucose

**Figure 1:**
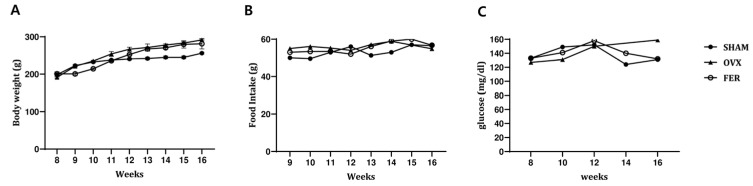
General physiological parameters in SHAM, OVX, and FER groups. (**A**) Body weight, (**B**) food intake, and (**C**) serum glucose levels were measured in SHAM, OVX, and FER groups over the experimental period. No significant differences were found among the groups in any of the physiological parameters measured. FER, ferrostatin-1-treated ovariectomized rats; OVX, ovariectomized rats; SHAM, sham-operated rats.

To examine the potential effects of ferrostatin-1 on systemic metabolic parameters in OVX rats, body weight, food intake, and serum glucose levels were monitored over an 8-week experimental period (Figure 1). No significant differences in body weight were observed between the SHAM and OVX groups at any time point, and ferrostatin-1 treatment did not result in a significant change in body weight compared with that of the OVX group. Food intake remained comparable among the three groups throughout the study period, with no statistically significant changes detected.

Serum glucose levels tended to be slightly elevated in the OVX group relative to those of the SHAM controls by the end of the experimental period, suggesting a mild trend toward impaired glucose metabolism. However, the increase was not statistically significant. Administration of ferrostatin-1 did not noticeably affect serum glucose levels in OVX rats. These findings indicate that ovariectomy in this model did not lead to overt metabolic dysregulation, and ferrostatin-1 treatment had no measurable impact on body weight, food intake, or glucose levels.

### 3.2. Lipogenesis and Salivary Lipid Accumulation in OVX Rats and Its Attenuation by Ferrostatin-1

**Figure 2:**
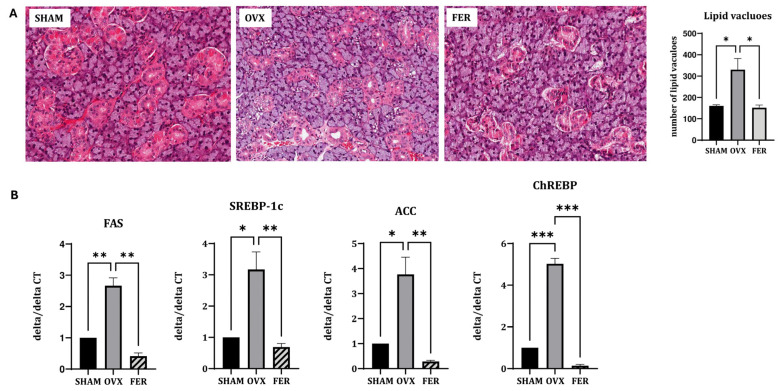
Lipogenesis and lipid metabolism in the salivary gland of SHAM, OVX, and FER groups. (**A**) Representative H&E-stained images. No significant morphological abnormalities were observed in any group. Lipid vacuole accumulation was increased in the OVX group compared to SHAM and reduced in the FER group. (**B**) Relative mRNA expression levels of lipogenic genes. The expression of all tested lipogenic genes was significantly increased in the OVX group compared to SHAM, which was attenuated by ferrostatin-1 treatment. * *p* < 0.05, ** *p* < 0.01, *** *p* < 0.001. Columns and error bars represent mean ± standard deviation. FER, ferrostatin-1-treated ovariectomized rats; H&E, hematoxylin and eosin; OVX, ovariectomized rats; SHAM, sham-operated rats.

We compared the histological and transcriptional changes associated with lipid metabolism in the salivary glands of OVX rats, with or without ferrostatin-1 treatment. H&E staining revealed that the overall salivary gland morphology, including the architecture of ducts and acinar cells, was preserved across all groups. No overt structural abnormalities were observed in the OVX or ferrostatin-1-treated (FER) groups compared with those of the SHAM group, suggesting that basic tissue integrity remained intact despite hormonal manipulation or treatment. In contrast, an increased number of cytoplasmic lipid vacuoles was observed in the salivary glands of OVX rats, indicating lipid accumulation. This histological alteration was markedly reduced in the ferrostatin-1-treated group, suggesting that ferroptosis inhibition may alleviate OVX-induced lipid dysregulation (Figure 2A).

Consistent with the histological findings, RT-qPCR demonstrated that the mRNA expression levels of lipogenic genes were significantly upregulated in the OVX group. Specifically, SREBP-1c expression increased approximately 2.8-fold in OVX rats and was significantly reduced in the ferrostatin-1-treated group (*p* < 0.05 and *p* < 0.01, respectively). ChREBP expression was elevated by 3.1-fold in the OVX group, and this induction was markedly attenuated in the FER group (*p* < 0.001). Similarly, *FAS* mRNA levels increased 2.7-fold in OVX rats and were significantly suppressed after ferrostatin-1 administration (*p* < 0.01). ACC expression also showed a 3.4-fold increase in the OVX group compared with that in SHAM, and this was significantly reduced by ferrostatin-1 (*p* < 0.05 and *p* < 0.01, respectively; Figure 2B). These results indicate that ferrostatin-1 effectively downregulated OVX-induced lipogenesis at the transcriptional level.

### 3.3. Ferroptosis-Associated Oxidative Stress Markers

**Figure 3:**
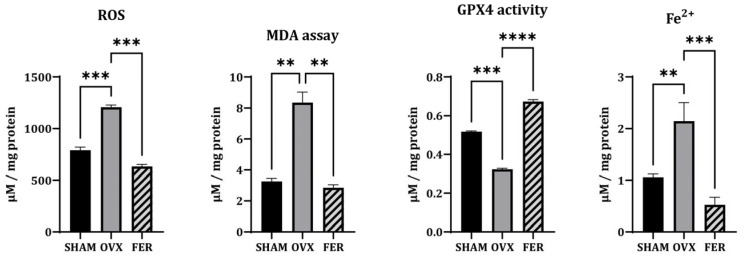
Oxidative stress markers associated with ferroptosis in the salivary glands of SHAM, OVX, and FER groups. Reactive oxygen species (ROS) levels, measured using DCF-DA fluorescence, were elevated in the OVX group and reduced in the FER group. Similarly, malondialdehyde (MDA) levels were increased in the OVX group and decreased following ferrostatin-1 treatment. GPX4 activity was reduced in the OVX group and restored in the FER group. In addition, cytosolic ferrous iron (Fe^2+^) levels were elevated in the OVX group and returned to baseline in the FER group. ** *p* < 0.01, *** *p* < 0.001, **** *p* < 0.0001. FER, ferrostatin-1-treated ovariectomized rats; GPX4, glutathione peroxidase 4; OVX, ovariectomized rats; SHAM, sham-operated rats.

Based on the observed lipid accumulation in OVX salivary glands, we examined whether ferroptosis, a form of lipid peroxidation-driven cell death, was involved in this process, and ferrostatin-1 could mitigate these changes. To assess ferroptosis, we evaluated ROS levels, MDA, ferrous iron (Fe^2+^), and the expression of GPX4 (Figure 3).

ROS levels, as measured by DCF-DA fluorescence intensity, were significantly elevated in OVX rats compared with those in SHAM controls, indicating increased oxidative stress. This increase was significantly lower in the ferrostatin-1-treated group (*p* < 0.001). Similarly, MDA levels, which reflect lipid peroxidation, were markedly higher in OVX rats than those in SHAM, and this lipid peroxidation was attenuated by ferrostatin-1 treatment (*p* < 0.01). In addition, cytosolic Fe^2+^ levels were elevated in the OVX group and normalized by ferrostatin-1 (*p* < 0.01 and *p* < 0.001, respectively), suggesting iron overload as a contributing factor to ferroptotic stress. Conversely, GPX4 activity was significantly reduced in OVX rats, which was reversed by ferrostatin-1 administration (*p* < 0.001).

These findings suggest that OVX-induced ferroptosis in the salivary glands is associated with ROS accumulation, lipid peroxidation, and iron overload, all of which are ameliorated by ferrostatin-1.

### 3.4. Ferroptosis-Driven Fibrosis and Inflammation and the Protective Role of Ferrostatin-1

Ferroptosis promotes fibrotic remodeling in various organs. To assess whether ferroptosis activation in OVX salivary glands contributes to fibrosis, we evaluated histological and molecular markers of fibrotic changes.

**Figure 4:**
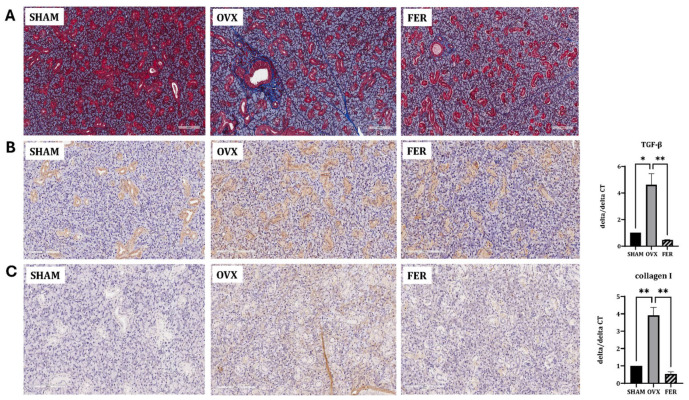
Assessment of fibrosis and inflammation in the salivary glands of SHAM, OVX, and FER groups. (**A**) Masson’s trichrome staining revealed increased collagen deposition in the OVX group compared with that in the SHAM group, which was reduced in the FER group. (**B**) Immunohistochemical and qPCR analyses for TGF-β1 revealed elevated expression in the OVX group, which was reduced in the FER group. (**C**) Collagen type I expression, assessed by immunohistochemistry and qPCR, was increased in the OVX group and attenuated following FER treatment. * *p* < 0.05, ** *p* < 0.01. Columns and error bars *represent* mean ± standard deviation. FER, ferrostatin-1-treated ovariectomized rats; OVX, ovariectomized rats; SHAM, sham-operated rats; qPCR, quantitative polymerase chain reaction.

Masson’s trichrome staining revealed marked collagen fiber deposition in the salivary glands of OVX rats compared with that in SHAM controls, predominantly surrounding ducts and acinar cells. This fibrotic accumulation was significantly attenuated in the ferrostatin-1-treated group, with the staining intensity and distribution more closely resembling that of the SHAM group (Figure 4A).

Immunohistochemical staining for TGF-β1 showed increased positive staining intensity in the OVX group, particularly in the periductal and periacinar regions, indicating enhanced fibrotic signaling. This increase was markedly attenuated in the ferrostatin-1-treated group (Figure 4B). Similarly, collagen type I staining was enhanced in OVX rats with a broad interstitial distribution and noticeably diminished following ferrostatin-1 administration (Figure 4C). These IHC findings were supported by the mRNA expression analysis. *TGF-β1* mRNA levels increased approximately 2.5-fold in the OVX group compared with those in the SHAM and were significantly suppressed by ferrostatin-1 (*p* < 0.05 and *p* < 0.01, respectively). Collagen type I mRNA (Col1a1) was also elevated by 3.2-fold in OVX rats and significantly reduced in the ferrostatin-1-treated group (*p* < 0.01).

These results indicate that OVX induces fibrotic remodeling in the salivary gland and ferrostatin-1 suppresses this process at both the protein and transcript levels.

### 3.5. Ferroptosis-Induced Functional Impairment and the Protective Effect of Ferrostatin-1

To determine whether OVX-induced ferroptosis affects salivary gland function, we assessed the expression of key functional markers, including aquaporin 5 (AQP5) and amylase 2A (AMY2A), using immunohistochemistry.

**Figure 5:**
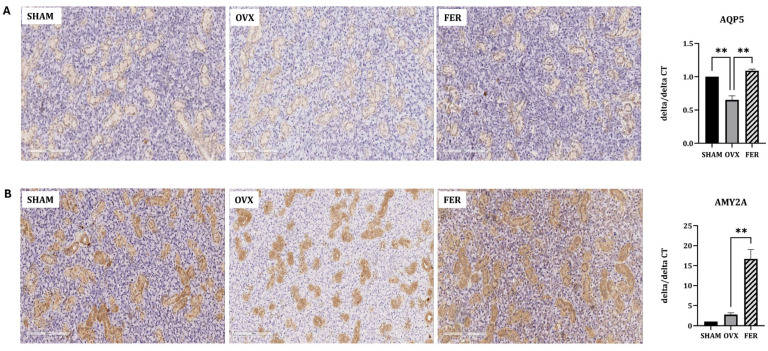
Expression of salivary gland functional proteins AQP5 and AMY2A in SHAM, OVX, and FER groups. (**A**) AQP5 expression was reduced in the OVX group and restored in the FER group, as shown by immunohistochemistry and qPCR. (**B**) AMY2A expression was decreased in the OVX group according to immunohistochemistry, and this reduction was prevented in the FER group. In the qPCR analysis, AMY2A levels in OVX were not significantly different from SHAM, whereas the FER group showed a significant elevation compared with SHAM. Together, these results indicate that ferrostatin-1 counteracts the OVX-induced reduction of AMY2A. ** *p* < 0.01. Columns and error bars represent mean ± standard deviation. FER, ferrostatin-1-treated ovariectomized rats; OVX, ovariectomized rats; SHAM, sham-operated rats; qPCR, quantitative polymerase chain reaction.

In SHAM rats, AQP5 was prominently localized to the apical membranes of ductal epithelial cells, indicating normal secretory function. In contrast, AQP5 expression markedly decreased in the OVX group, with diffuse and reduced staining intensities. Notably, ferrostatin-1 treatment restored AQP5 expression, with staining patterns and intensities comparable to those of the SHAM group (Figure 5A). Similarly, AMY2A staining was clearly observed in the cytoplasm of acinar cells in the SHAM rats, reflecting an intact enzyme-producing capacity. This expression was significantly reduced in OVX rats, which was consistent with compromised glandular function. Treatment with ferrostatin-1 recovered AMY2A expression, restoring cytoplasmic localization and staining intensity (Figure 5B). In line with the IHC findings, quantitative real-time PCR analysis revealed that the mRNA levels of AQP5 and AMY2A were significantly decreased in the OVX group and restored in the FER group, further supporting the protective role of ferrostatin-1 in preserving salivary gland function at both the transcriptional and protein levels (Figure 5).

These results suggest that ferroptosis contributes to functional impairment of the salivary gland in OVX rats, and ferrostatin-1 can preserve secretory and enzymatic functions by protecting against ferroptosis-induced damage.

### 3.6. The Effect of Ferrostatin-1 on Mitochondrial Antioxidant Defense

**Figure 6:**
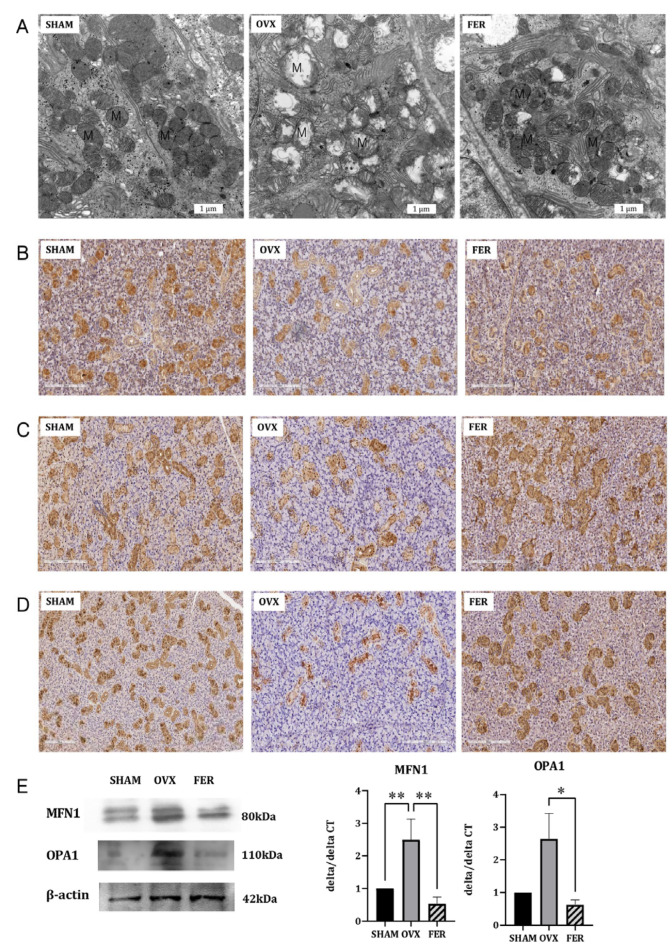
Effects of ferrostatin-1 on mitochondrial structure, antioxidant defense, and fusion dynamics in the salivary glands of SHAM, OVX, and FER groups. (**A**) Transmission electron microscopy (TEM) images showing preserved mitochondrial structure in SHAM, disrupted cristae and condensed matrix in OVX, and improved morphology in the FER group. Immunohistochemical staining for PGC-1α (**B**), SOD (**C**), and catalase (**D**) demonstrating reduced expression in OVX glands and recovery in the FER group. (**E**) Western blot and qPCR analyses of MFN1 and OPA1 showing decreased expression in the OVX group, which was restored by ferrostatin-1. * *p* < 0.05, ** *p* < 0.01. Columns and error bars represent mean ± standard deviation. FER, ferrostatin-1-treated ovariectomized rats; OVX, ovariectomized rats; SHAM, sham-operated rats; qPCR, quantitative polymerase chain reaction.

Given that ferroptosis is mechanistically linked to mitochondrial dysfunction and ferrostatin-1 is a known ferroptosis inhibitor, we investigated whether ferrostatin-1 confers mitochondrial protection to the salivary glands of OVX rats. Transmission electron microscopy (TEM) revealed substantial mitochondrial damage in the OVX rats, including a condensed matrix and disrupted cristae, both of which are characteristic ultrastructural features of ferroptotic injury. In contrast, mitochondria in ferrostatin-1-treated rats retained their normal morphology, indicating preserved mitochondrial integrity (Figure 6A).

We further evaluated mitochondrial antioxidant defense by immunohistochemical staining for PGC-1α, superoxide dismutase (SOD), and catalase. PGC-1α, a master regulator of mitochondrial biogenesis, was markedly reduced in OVX salivary glands, especially in ductal and acinar cells, and was substantially restored by ferrostatin-1 (Figure 6B). Similarly, both SOD and catalase expression were downregulated in OVX rats, but recovered to near-SHAM levels following ferrostatin-1 treatment (Figure 6C,D). Consistent with the IHC results, qPCR analysis showed that the mRNA levels of PGC-1α, SDD, and catalase were significantly reduced in the OVX group and restored in the FER group (Supplementary Figure S1). These findings collectively indicate that ferrostatin-1 enhances the mitochondrial antioxidant defense capacity in OVX salivary glands.

To assess whether mitochondrial dynamics were also affected, we measured MFN1 and OPA1 expression using Western blotting and qPCR (Figure 6E). MFN1 expression was significantly decreased at both the mRNA and protein levels in OVX rats compared with that in SHAM controls, and this reduction was partially reversed by ferrostatin-1 treatment (*p* < 0.01). OPA1 expression was also significantly downregulated in the OVX group and ferrostatin-1 administration restored its levels to those in the SHAM group (*p* < 0.05).

Taken together, these results demonstrate, for the first time, that ferrostatin-1 mitigates mitochondrial oxidative injury and dysfunction in OVX salivary glands by improving antioxidant capacity, biogenesis, and dynamics. This represents a key mechanistic distinction from our previous studies and positions mitochondria as a central regulatory node in ferroptosis-driven salivary gland dysfunction.

### 3.7. Mitophagy-Associated Ferroptosis in OVX Salivary Glands

**Figure 7:**
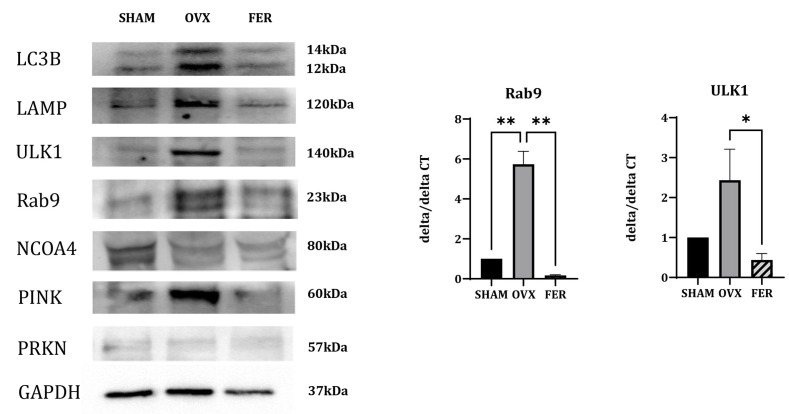
Expression of mitophagy- and autophagy-related markers in the salivary glands of SHAM, OVX, and FER groups. Immunoblotting and qPCR were used to examine expression levels of mitophagy- and autophagy-related markers. Protein expression of PINK1 was increased in the OVX group and reduced in the FER group. ULK1 expression was elevated in the OVX group and appeared decreased in the FER group at both the protein and mRNA levels. Rab9 levels were also upregulated in the OVX group and decreased following ferrostatin-1 treatment, as shown by both Western blotting and qPCR. No significant change in PRKN expression was observed among the three groups. LAMP and LC3B expression was increased in OVX rats and reduced in the FER group. NCOA4 expression showed no notable difference among the groups. All data are representative of at least three independent experiments. * *p* < 0.05, ** *p* < 0.01. Columns and error bars represent mean ± standard deviation. FER, ferrostatin-1-treated ovariectomized rats; OVX, ovariectomized rats; SHAM, sham-operated rats; qPCR, quantitative polymerase chain reaction.

To explore the potential autophagy-related mechanisms contributing to ferroptosis in OVX salivary glands, we analyzed the expression of genes involved in various forms of selective autophagy (Figure 7).

The expression of NCOA4, a selective cargo receptor that mediates ferritin degradation via ferritinophagy, did not significantly differ between the SHAM, OVX, and ferrostatin-1-treated groups, suggesting that ferritinophagy is not prominently involved in this context.

In contrast, the expression of multiple mitophagy-related markers was altered. PINK1, a mitochondrial serine/threonine kinase that senses depolarized mitochondria and initiates mitophagy, showed increased protein expression in the OVX group, which was reduced by ferrostatin-1 treatment.

ULK1, which initiates autophagosome formation in both general autophagy and mitophagy, was upregulated at both the mRNA and protein levels in OVX rats and reduced in the ferrostatin-1-treated group. Rab9, a small GTPase involved in receptor-independent, alternative mitophagy, showed increased expression in OVX rats in both qPCR and Western blotting, and was downregulated by ferrostatin-1. PRKN (Parkin), an E3 ubiquitin ligase typically recruited by PINK1, showed no appreciable changes among the three groups, suggesting that it may not be significantly involved in this model.

Additional autophagy-related markers were assessed. LAMP, a lysosomal membrane protein essential for autophagosome-lysosome fusion, was upregulated in the OVX group and appeared to decrease following ferrostatin-1 treatment. LC3B, a core component of the autophagosomal membrane and general marker of autophagosome formation, showed increased expression in OVX rats and reduced to near-SHAM levels in the ferrostatin-1 group.

These findings indicate that ferroptosis in OVX salivary glands is associated with enhanced mitophagy, particularly through PINK1-dependent and alternative pathways involving ULK1 and Rab9, rather than ferritinophagy. Mitophagy activation was accompanied by the upregulation of LAMP and LC3B and appeared to be suppressed by ferrostatin-1. These results differ from those of our previous liver-based model and highlight a distinct, organ-specific ferroptotic mechanism.

## 4. Discussion

In this study, we demonstrated that ferroptosis plays a crucial role in the salivary gland dysfunction induced by estrogen deficiency in an ovariectomized (OVX) rat model. Notably, we found that the early administration of ferrostatin-1 attenuated salivary gland injury by inhibiting oxidative damage, fibrosis, and inflammatory responses. Unlike previous studies that mainly focused on classical markers of ferroptosis, such as ROS accumulation and GPX4 suppression, our data suggest that mitochondrial quality control, particularly mitophagy and fusion-related gene regulation, may serve as an upstream modulator of ferroptosis in this model [18,19]. These findings provide novel insights into the pathophysiological mechanisms underlying menopause-associated salivary gland dysfunction and highlight a potential preventive role for ferroptosis inhibitors.

Our previous reports, as well as other studies, have established that ferroptosis contributes to tissue damage under estrogen-deficient conditions [12,13,20,21,22]. In our earlier studies using OVX models, we showed that ferroptosis is associated with salivary gland inflammation, fibrosis, and functional impairment and that ferrostatin-1 alleviates these effects. However, these investigations focused primarily on the terminal events of ferroptosis, such as lipid peroxidation and GPX4 depletion, without addressing the upstream regulatory networks that initiate this form of cell death. The current study expands on this foundation by identifying mitochondrial dysfunction and mitophagy as potential upstream modulators of ferroptosis. Notably, NCOA4-mediated ferritinophagy was previously shown to be a key driver of ferroptosis in the liver tissue [14]. However, our findings indicate that this pathway is not activated in the salivary glands of OVX rats. Instead, we observed significant alterations in mitophagy-related markers, such as PINK1, ULK1, and Rab9, suggesting possible tissue-specific divergence in the mechanisms of ferroptosis regulation. These organ-specific differences in ferroptotic control mechanisms underscore the importance of contextualizing ferroptosis within the cellular and metabolic environments of each tissue type.

These findings provide novel insights into the mechanistic relationship between mitochondrial homeostasis and ferroptosis in estrogen-deficient salivary glands. Consistent with our previous work [12,13], we confirmed that ferrostatin-1 treatment attenuated OVX-induced salivary gland fibrosis, inflammation, and secretory dysfunction, as demonstrated by histological staining, reduced expression of profibrotic and inflammatory markers (TGF-β1, Col1a1, TNF-α, and IL-6), and preservation of secretory proteins, such as AQP5 and AMY2A. Importantly, this study further revealed that the OVX model exhibited significant mitochondrial damage, as evidenced by electron microscopy and reduced expression of antioxidant defense markers, including PGC-1α, SOD, and catalase. These changes were accompanied by impaired expression of mitochondrial fusion-related genes (*MFN1* and *OPA1*), suggesting that the disruption of mitochondrial integrity may serve as a critical early event in ferroptosis initiation.

Mitophagy-related markers, including PINK1, ULK1, and Rab9, were upregulated in the OVX group and attenuated by ferrostatin-1 treatment. This implies that estrogen deficiency triggers compensatory activation of mitophagy to eliminate damaged mitochondria; however, sustained activation may paradoxically exacerbate ferroptotic cell death by depleting healthy mitochondria and promoting ROS accumulation. These findings suggest that mitochondrial dysfunction, rather than ferritin turnover, plays a central role in ferroptosis in estrogen-deficient salivary glands.

Taken together, our data indicate that ferroptosis in postmenopausal salivary glands is likely driven not only by iron overload and lipid peroxidation, but also by dysregulated mitophagy and compromised mitochondrial antioxidant defenses. This integrated mechanism provides a more comprehensive understanding of how mitochondrial instability serves as a critical upstream trigger for ferroptosis under estrogen-deficient conditions.

Ferroptosis is a universal cell death pathway characterized by iron-dependent lipid peroxidation [18,19,23,24]. However, growing evidence suggests that its regulatory mechanisms exhibit significant organ-specific variations depending on tissue type, cellular metabolism, and iron homeostasis [15,17,25,26]. We previously found that NCOA4-mediated ferritinophagy plays a central role in modulating ferroptosis in hepatic tissues [14]. In contrast, the current study demonstrated no detectable change in NCOA4 expression in estrogen-deficient salivary glands, but rather a pronounced upregulation of mitophagy-associated markers, such as PINK1, ULK1, and Rab9. These findings highlight the divergence in upstream ferroptosis regulation, where mitochondrial degradation through mitophagy may serve as the dominant mechanism in certain tissues, whereas iron sequestration via ferritinophagy prevails in others. This tissue specificity may reflect the differences in mitochondrial content, iron storage capacity, or antioxidant buffering systems across organs. Similar distinctions have been reported in other contexts, such as lipid metabolism-driven ferroptosis in adipose tissue, and GPX4-centered regulation in neural and renal tissues, underscoring the complexity of ferroptosis as a cell death program that is not uniform across biological systems [15,17,26,27]. Thus, our data support the concept that ferroptosis is a context-dependent process and understanding its organ-specific regulatory nodes is critical for the development of targeted therapeutic strategies. In the case of salivary glands, the prominent involvement of mitophagy rather than ferritinophagy may have implications for the design of ferroptosis inhibitors tailored to glandular tissue or menopausal conditions.

**Figure 8:**
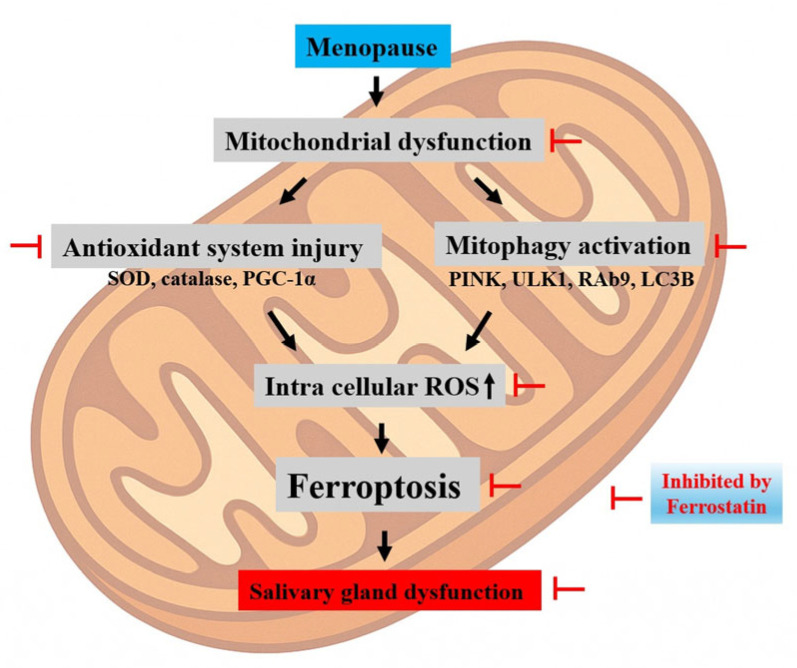
Proposed mechanism of mitophagy-driven ferroptosis in estrogen-deficient salivary glands. A schematic illustration depicting how mitochondrial dysfunction initiates a cascade involving antioxidant system injury, mitophagy activation, and ferroptosis, ultimately leading to salivary gland dysfunction in OVX rats. Key molecular changes include decreased expression of PGC-1α, SOD, and catalase, impaired mitochondrial dynamics (MFN1 and OPA1), and upregulation of mitophagy-related genes (PINK1, ULK1, Rab9, and LC3B). This pathological sequence is inhibited by ferrostatin-1.

In this study, early administration of ferrostatin-1 effectively prevented the onset of salivary gland dysfunction in OVX rats, reinforcing its potential as a preventive strategy rather than a mere therapeutic intervention for already damaged tissues. Ferrostatin-1, a lipid peroxidation inhibitor, is known to neutralize ROS and suppress lipid ROS accumulation, thereby mitigating ferroptotic cell death [28,29,30,31]. However, our findings suggest that these protective effects extend to the preservation of mitochondrial integrity. Specifically, ferrostatin-1 treatment restored the expression of key mitochondrial fusion regulators, such as MFN1 and OPA1, and upregulated antioxidant defense components. These changes indicate that ferrostatin-1 not only suppresses downstream ferroptotic execution but also stabilizes upstream mitochondrial dynamics and redox balance, which are often disrupted in estrogen-deficient states. Moreover, the attenuation of mitophagy-related gene expression following ferrostatin-1 treatment implies that this agent may indirectly suppress maladaptive mitophagy, which fuels mitochondrial instability and ferroptosis [25,32,33,34,35,36]. The proposed mechanistic cascade is summarized in Figure 8, which highlights how mitochondrial dysfunction initiates a pathological sequence involving antioxidant system injury (e.g., decreased SOD, catalase, and PGC-1α), activation of mitophagy (e.g., PINK1, ULK1, Rab9, and LC3B), and accumulation of intracellular ROS. This culminates in ferroptotic cell death, ultimately leading to salivary gland dysfunction. Ferrostatin-1 disrupts this pathogenic sequence by intervening at multiple levels, particularly by preserving mitochondrial homeostasis and blocking ferroptotic signaling. Taken together, these results suggest that ferrostatin-1 is not only a ferroptosis blocker, but also a modulator of mitochondrial health and cellular homeostasis. This expands its therapeutic implications beyond ferroptosis suppression toward the broader maintenance of mitochondrial resilience, especially in hormone-deficient or aging-related contexts, such as menopause.

Importantly, this study substantially extends our previous findings by exploring upstream regulatory pathways that were not investigated in earlier work. While our prior study focused on terminal ferroptosis markers and the protective effects of ferrostatin-1 at the histological and biochemical level, it did not address the upstream mitochondrial quality control systems that initiate ferroptosis. In contrast, the current study integrates multiple layers of evidence—including mitophagy marker expression, mitochondrial antioxidant regulation, and fusion dynamics—to elucidate the initiation cascade of ferroptosis in estrogen-deficient salivary glands. This mechanistic depth represents a significant advancement beyond our previous work and provides a more comprehensive framework for understanding the organ-specific regulation of ferroptosis in menopausal conditions.

Despite this comprehensive analysis, this study has several limitations. First, although mitophagy and mitochondrial antioxidant failure were implicated in ferroptosis regulation, the findings remain correlative. Functional validation using genetic or pharmacological modulation of mitophagy-related genes (*PINK1*, *PRKN*, and *ULK1*) is required to establish causality. Second, the investigation was limited to the submandibular glands of female Sprague–Dawley rats. Whether similar mechanisms are involved in other salivary glands or species remains uncertain, and the absence of in vitro or human tissue data restricts their translational relevance. In particular, given the known structural and metabolic differences among various salivary glands (e.g., parotid, sublingual, and minor glands), the susceptibility to ferroptotic injury may vary depending on gland type. Third, although immunohistochemistry and Western blotting revealed significant molecular alterations, the lack of quantitative protein analysis and mitochondrial functional assays may limit the precision of the mechanistic interpretation.

## 5. Conclusions

This study provides novel insights into organ-specific regulation of ferroptosis in estrogen-deficient salivary glands. Unlike our previous findings in hepatic tissue, where ferritinophagy was the central driver, ferroptosis in the postmenopausal salivary glands appears to be more closely associated with impaired mitophagy and mitochondrial antioxidant failure. Through the early administration of ferrostatin-1, we successfully demonstrated the suppression of ferroptosis and its downstream consequences, including inflammation, fibrosis, and secretory dysfunction.

These findings expand the understanding of ferroptotic pathways in salivary tissue and suggest that targeting mitochondrial stability and mitophagy may represent a promising preventive strategy against menopause-induced glandular degeneration. Further studies are needed to validate these mechanisms and explore their therapeutic relevance in clinical settings.

## Figures and Tables

**Table 1:** Primer sequences used for polymerase chain reaction analysis.

Gene	Accession NO.	Sequence (5′–3′)	
		Forward	Reverse
*GAPDH*	NM_017008	GCAAGTTCAACGGCACAG	CGCCAGTAGACTCCACGAC
*SREBP-1c*	NM_001276707.1	TGCCCTAAGGGTCAAAACCA	TGGCGGGCACTACTTAGGAA
*ChREBP*	NM_133552	CAGATGCGGGACATGTTTGA	AATAAAGGTCGGATGAGGATGCT
*FAS*	NM_017332	CTTGGGTGCCGATTACAACC	GCCCTCCCGTACACTCACTC
*ACC*	NM_017332.2	AGGAAGATGGTGTCCCGCTCTG	GGGGAGATGTGCTGGGTCAT
*TGF-β1*	NM_021578.2	ACTACGCCAAAGAAGTCACCC	GCCCTGTATTCCGTCTCCTT
*CollagenI*	NM_053304.1	TGGATGGCTGCACGAGT	TTGGGATGGAGGGAGTTTA
*AQP5*	NM_012779.1	CATGAACCCAGCCCGATCTT	AGAAGACCCAGTGAGAGGGG
*AMY2A*	NM_031502	TTTGGCAGAGGAAACAAAGGC	TGACATCACAGTATGTGCCAGC
*PGC-1α*	NM_031347.1	GTGCAGCCAAGACTCTGTATGG	GTCCAGGTCATTCACATCAAGTTC
*SOD*	NM_053304.1	TTTTGCTCTCCCAGGTTCCG	CCCATGCTCGCCTTCAGTTA
*catalase*	NM_214301.2	CTTGGAACATTGTACCCGCT	GTCCAGAAGAGCCTGAATGC
*OPA1*	NM_001433907	TCTCAGCCTTGCTGTGTCAGAC	TTCCGTCTCTAGGTTAAAGCGCG
*MFN1*	NM_001244768.1	CCAGGTACAGATGTCACCACAG	TTGGAGAGCCGCTCATTCACCT

## Data Availability

All of the data is contained within the article and Supplementary Materials.

## Supplementary Materials

The following supporting information can be downloaded at: https://www.mdpi.com/article/10.3390/antiox14091058/s1, Figure S1. Relative mRNA expression levels of PGC-1α, SOD, and catalase in salivary glands from the SHAM, OVX, and FER groups, analyzed by qPCR.
