# Supplementary material for: Ferrostatin-1 Prevents Salivary Gland Dysfunction in an Ovariectomized Rat Model by Suppressing Mitophagy-Driven Ferroptosis
Source: Antioxidants (Basel). 2025 Aug 28;14(9):1058. doi: 10.3390/antiox14091058 (PMC12466376; doi:10.3390/antiox14091058)
Supplement: Supplementary file 1 [file antioxidants-14-01058-s001.zip › antioxidants-3745076-supplementary.pdf]

Supplementaty Figure S1

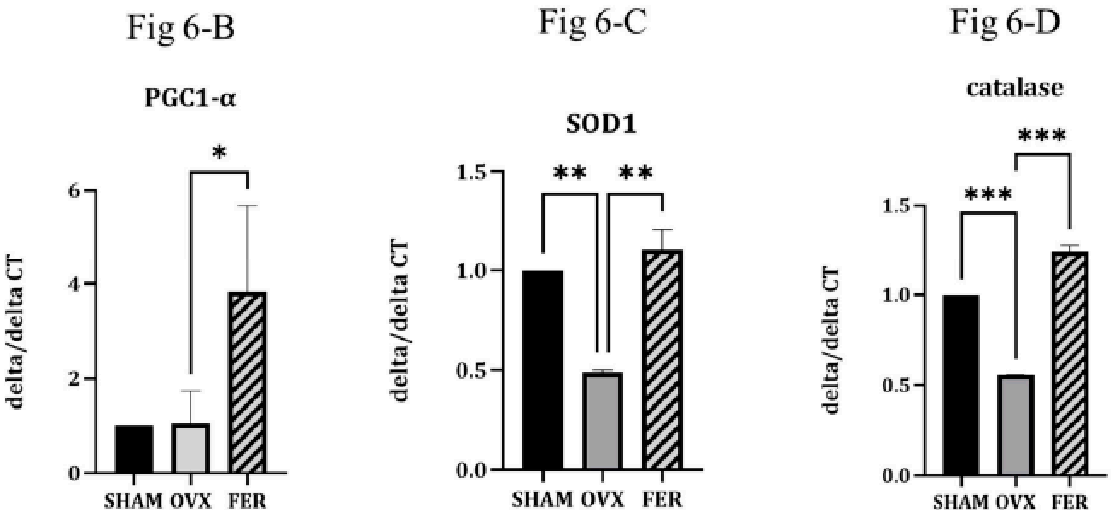

Figure S1. Relative mRNA expression levels of PGC-1*α*, SOD, and catalase in salivary glands from the SHAM, OVX, and FER groups, analyzed by qPCR. The expression of all three genes was significantly reduced in the OVX group and restored by ferrostatin-1 treatment, consistent with the immunohistochemistry results. Data are presented as mean ± SD. *$p < 0.05$, **$p < 0.01$, ***$p < 0.001$.
